# Supplementary material for: Parental kynurenine 3-monooxygenase genotype in mice directs sex-specific behavioral outcomes in offspring
Source: Biol Sex Differ. 2025 Apr 2;16:22. doi: 10.1186/s13293-025-00703-w (PMC11967062; doi:10.1186/s13293-025-00703-w)

*TITLE:* **Parental kynurenine 3-monooxygenase genotype in mice directs sex-specific behavioral outcomes in offspring**

**Authors:** Snezana Milosavljevic^1^, Maria V. Piroli^1^, Emma J. Sandago^1^, Gerardo G. Piroli^1^, Holland H. Smith^1^, Norma Frizzell^1^, Sarah Beggiato^2^, Ana Pocivavsek^1^

**Affiliation:**

^1^Department of Pharmacology, Physiology and Neuroscience, University of South Carolina School of Medicine, Columbia, South Carolina

^2^Department of Life Sciences and Biotechnology, University of Ferrara, Ferrara, Italy

**Corresponding Author:**

Ana Pocivavsek, Ph.D.

University of South Carolina School of Medicine

Department of Pharmacology, Physiology and Neuroscience

Building 1, D26

6311 Garners Ferry Rd

Columbia, SC 29209, USA

Phone: (803) 216-3509

[ana.pocivavsek@uscmed.sc.edu](mailto:ana.pocivavsek@uscmed.sc.edu)

**Supplemental Statistical Table.** All three-way and two-way ANOVA statistical results are presented in the supplemental .xls file named “*Milosavljevic et al_Supplemental Statistical Table*” to denote statistical analysis for manuscript figures and supplemental figures wherein male and female data are evaluated simultaneously. Sample size for each experiment is also provided in this statistical table.

**Supplementary Table 1. Sleep-wake parameters in HET-*Kmo^+/-^* mice in comparison to wild-type (WT-Control) mice during the dark phase.** Data are mean ± SEM. Unpaired t test: *P<0.05 vs. WT-Control. N = 6-8 per group.

| **Dark Phase** | **Female** | | **Male** | |
| --- | --- | --- | --- | --- |
|  | WT-Control | HET-*Kmo^+/-^* | WT-Control | HET-*Kmo^+/-^* |
| REM Duration (% total time) | 2.4 ± 0.1 | 2.2 ± 0.3 | 2.9 ± 0.2 | 2.8 ± 0.2 |
| NREM Duration (% total time) | 34 ± 3 | 31 ± 4 | 36 ± 2 | 34 ± 2 |
| Wake Duration (% total time) | 63 ± 3 | 66 ± 4 | 61 ± 2 | 64 ± 2 |
| REM Bouts (#) | 15 ± 1 | 15 ± 1 | 18 ± 1 | 20 ± 2 |
| NREM Bouts (#) | 71 ± 5 | 87 ± 12 | 92 ± 6 | 82 ± 6 |
| Wake Bouts (#) | 74 ± 5 | 90 ± 11 | 93 ± 6 | 85 ± 6 |
| REM Bout Duration (s/bout) | 77 ± 5 | ***65 ± 2 **** | 72 ± 3 | 64 ± 3 |
| NREM Bout Duration (s/bout) | 223 ± 24 | 167 ± 8 | 178 ± 12 | 187 ± 10 |
| Wake Bout Duration (s/bout) | 769 ± 92 | 908 ± 230 | 619 ± 127 | 631 ± 81 |
| Relative Cage Activity (sum) | 2964 ± 443 | 3304 ± 662 | 2227 ± 288 | 2512 ± 325 |

**Supplementary Table 2. Sleep onset and number of transitions between vigilance states in female and male mice during light and dark phase.** Data are mean ± SEM. Unpaired t test: *P<0.05 vs. WT-Control. N = 6-10 per group.

| **Light Phase** | **Female** | | |
| --- | --- | --- | --- |
|  | WT-Control | WT-*Kmo^+/+^* | HET-*Kmo^+/-^* |
| NREM Onset (min) | 4.9 ± 1.8 | 6.7 ± 1.2 | 12.2 ± 3.8 |
| REM Onset (min) | 29.3 ± 8.3 | 23.9 ± 4.2 | 35.3 ± 6.0 |
| Wake to NREM (#) | 102.8 ± 6.3 | 107.5 ± 5.9 | ***124.8 ± 6.8 **** |
| NREM to REM (#) | 34.7 ± 2.4 | 30.3 ± 2.1 | 32.3 ± 3.0 |
| REM to Wake (#) | 32.9 ± 2.1 | 29.1 ± 1.9 | 31.6 ± 3.1 |
| NREM to Wake (#) | 70.1 ± 4.6 | 78.3 ± 5.5 | ***92.8 ± 8.9 **** |
| REM to NREM (#) | 1.8 ± 0.4 | 1.2 ± 0.4 | ***0.6 ± 0.2 **** |
| Total Transitions (#) | 242.3 ± 15.3 | 246.4 ± 12.4 | 282.1 ± 12.4 |

| **Light Phase** | **Male** | | |
| --- | --- | --- | --- |
|  | WT-Control | WT-*Kmo^+/+^* | HET-*Kmo^+/-^* |
| NREM Onset (min) | 6.1 ± 3.0 | 4.6 ± 2.9 | ***15.3 ± 2.8 **** |
| REM Onset (min) | 27.1 ± 3.9 | 20.4 ± 4.1 | 29.2 ± 3.8 |
| Wake to NREM (#) | 112.9 ± 8.1 | 99.1 ± 2.9 | 108.4 ± 6.8 |
| NREM to REM (#) | 34.9 ± 2.2 | 33.3 ± 1.9 | 36.8 ± 2.3 |
| REM to Wake (#) | 32.8 ± 2.1 | 32.1 ± 1.7 | 35.4 ± 2.4 |
| NREM to Wake (#) | 80.1 ± 7.8 | 67.3 ± 3.8 | 72.8 ± 7.6 |
| REM to NREM (#) | 2.1 ± 0.3 | 1.2 ± 0.4 | 1.4 ± 0.4 |
| Total Transitions (#) | 262.9 ± 16.8 | 232.9 ± 5.3 | 254.9 ± 12.9 |

| **Dark Phase** | **Female** | | |
| --- | --- | --- | --- |
|  | WT-Control | WT-*Kmo^+/+^* | HET-*Kmo^+/-^* |
| NREM Onset (min) | 13.0 ± 4.3 | 13.1 ± 4.4 | 12.0 ± 6.9 |
| REM Onset (min) | 44.9 ± 6.0 | 49.4 ± 13.9 | 59.2 ± 12.5 |
| Wake to NREM (#) | 66.5 ± 5.1 | 68.3 ± 5.7 | 82.1 ± 11.6 |
| NREM to REM (#) | 14.3 ± 1.0 | 16.5 ± 1.0 | 14.8 ± 1.5 |
| REM to Wake (#) | 14.0 ± 0.9 | 16.1 ± 0.9 | 14.3 ± 1.5 |
| NREM to Wake (#) | 52.5 ± 4.9 | 52.2 ± 5.0 | 68.0 ± 11.4 |
| REM to NREM (#) | 0.3 ± 0.2 | 0.3 ± 0.1 | 0.5 ± 0.2 |
| Total Transitions (#) | 147.7 ± 10.3 | 153.4 ± 12.3 | 179.6 ± 23.5 |

| **Dark Phase** | **Male** | | |
| --- | --- | --- | --- |
|  | WT-Control | WT-*Kmo^+/+^* | HET-*Kmo^+/-^* |
| NREM Onset (min) | 11.7 ± 4.2 | 8.1 ± 2.6 | 13.1 ± 6.6 |
| REM Onset (min) | 101.3 ± 23.4 | 92.2 ± 14.1 | 77.5 ± 14.6 |
| Wake to NREM (#) | 86.5 ± 5.9 | 75.3 ± 4.7 | 77.6 ± 6.0 |
| NREM to REM (#) | 18.1 ± 1.0 | 18.3 ± 1.0 | 19.7 ± 2.0 |
| REM to Wake (#) | 17.6 ± 0.9 | 18.1 ± 0.9 | 19.0 ± 1.9 |
| NREM to Wake (#) | 69.0 ± 5.8 | 57.1 ± 4.5 | 58.7 ± 6.1 |
| REM to NREM (#) | 0.5 ± 0.2 | 0.2 ± 0.1 | 0.7 ± 0.2 |
| Total Transitions (#) | 191.8 ± 12.0 | 168.9 ± 9.8 | 175.6 ± 12.3 |

**Supplementary Figure 1. HET-*Kmo^+/-^* mice have altered REM and NREM sleep power spectra in comparison to wild-type (WT-Control) mice during the dark phase.**

**(A)** REM sleep spectral power (Females: Two-way RM ANOVA Genotype x Frequency interaction F_(38, 380)_= 2.277, ^^^^P<0.0001; Males: Two-way RM ANOVA Genotype x Frequency interaction F_(38, 532)_= 2.765, ^^^^P<0.0001).

**(B)** NREM sleep spectral power (Females: Two-way RM ANOVA Genotype x Frequency interaction F_(38, 380)_= 2.455, ^^^^P<0.0001; Males: Two-way RM ANOVA Genotype x Frequency interaction F_(38, 532)_= 4.874, ^^^^P<0.0001). Data are mean ± SEM. N = 6-8 per group.


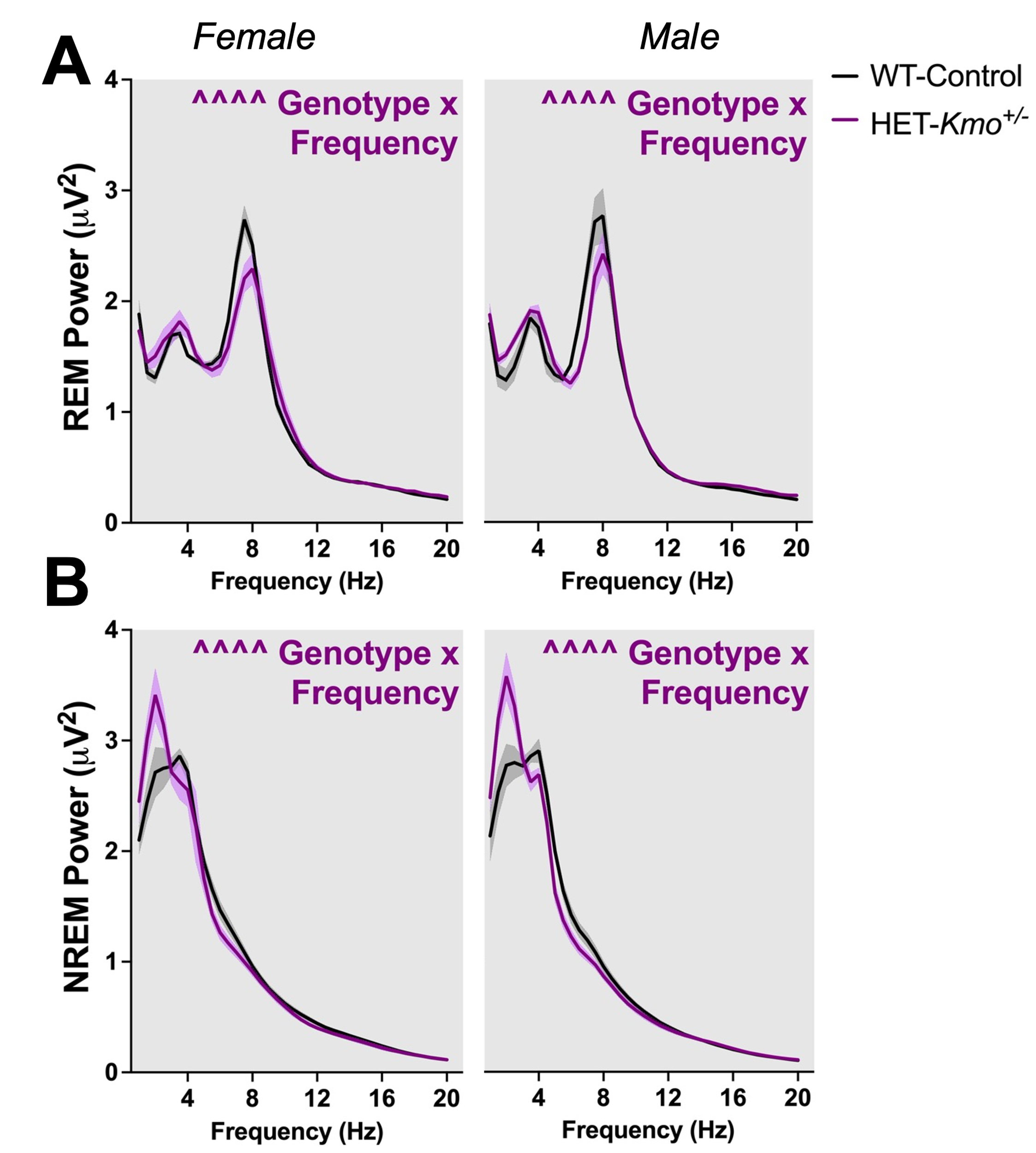
**Supplementary Figure 2. Brain content of 3-hydroxykynurenine is decreased in HET-*Kmo^+/-^* compared to WT-Control female mice.**

**(A)** Western blot profiling of VDAC2, an outer mitochondrial membrane marker, and HNE adducts, product of lipid peroxidation, in both the cortex and hippocampus of HET-*Kmo^+/-^* female mice and WT-Control.

**(B)** HNE adducts in the cortex.

**(C)** HNE adducts in the hippocampus.

**(D)** 3-hydroxykynurenine (3-HK) in the brain homogenates.

**(E)** VDAC2 in the cortical and hippocampal mitochondria.


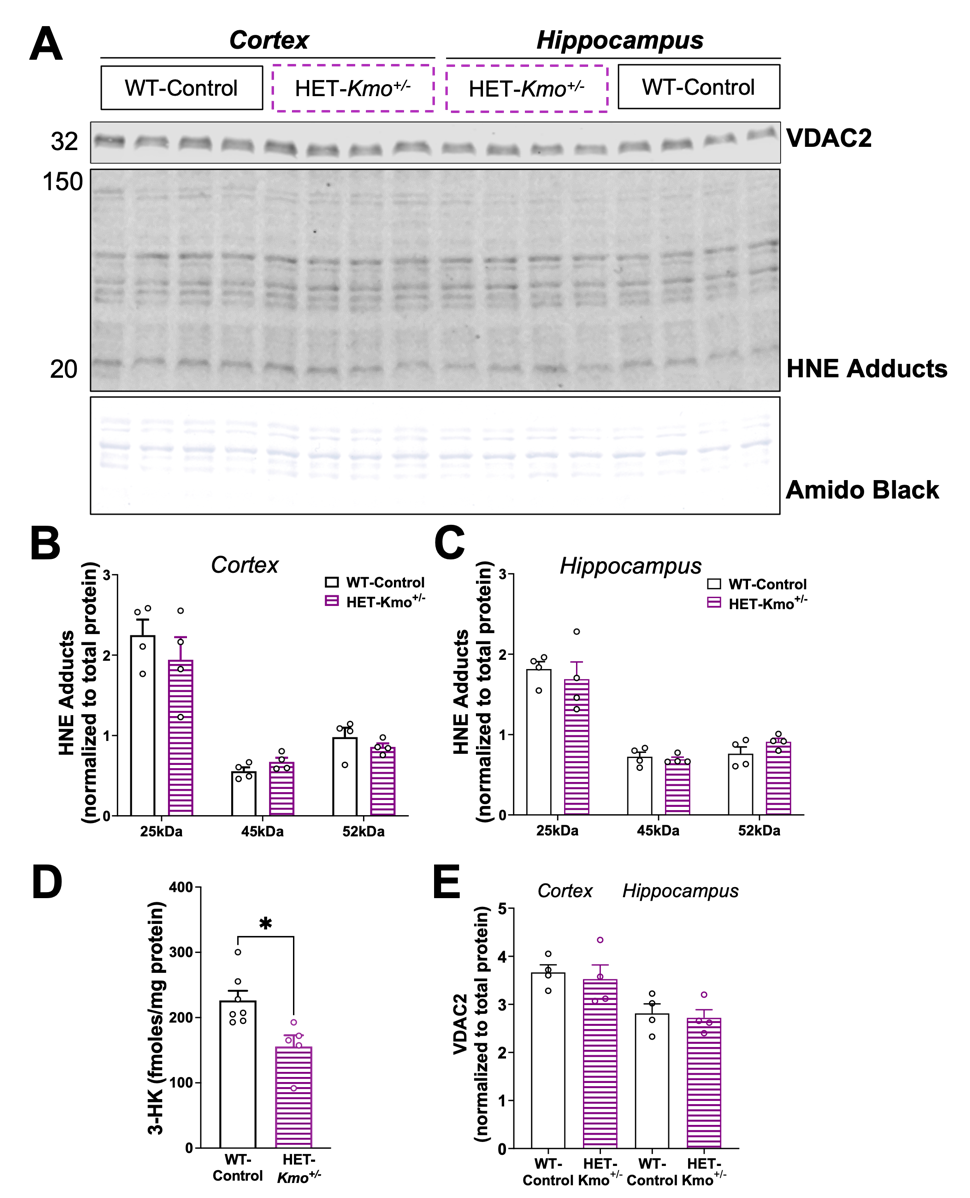
Data are mean ± SEM. Unpaired t test: **P<0.01. N = 4-7 per group.**Supplementary Figure 3. Parental behavior in HET-*Kmo^+/-^* mice in comparison to wild-type (WT-Control) mice.**

**(A)** Female parent behavior during early and late light phase.

**(B)** Male parent behavior during early and late light phase.

N = 7-10 per group.

**
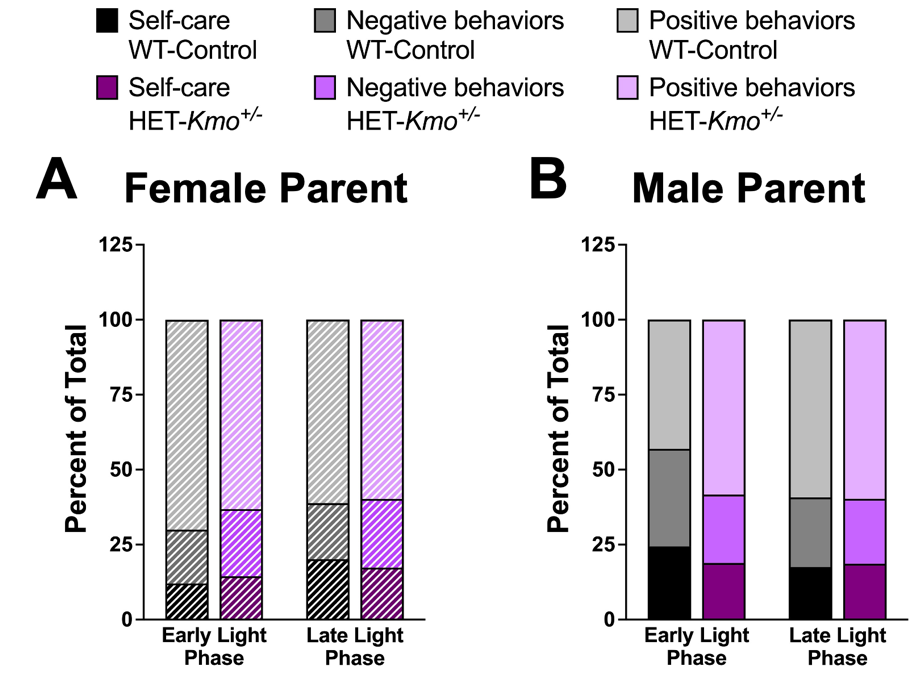
**

**Supplementary Table 3. Maternal and paternal behaviors in HET-*Kmo^+/-^* mice in comparison to wild-type (WT-Control) mice during early and late light phase.** Data are mean ± SEM. N = 7-10 per group.

| **Female Parent Behaviors** | | **Positive Behaviors** | | |
| --- | --- | --- | --- | --- |
| # of observations | | *Grooming pups* | *Nursing* | *Contact with pups* |
| *Early Light Phase* | **WT-Control** | 1.3 ± 0.2 | 7.0 ± 0.5 | 7.8 ± 0.6 |
|  | **HET-*Kmo^+/-^*** | 1.5 ± 0.2 | 6.6 ± 0.8 | 7.5 ± 0.5 |
| *Late Light Phase* | **WT-Control** | 1.3 ± 0.1 | 6.5 ± 0.2 | 7.7 ± 0.4 |
|  | **HET-*Kmo^+/-^*** | 1.8 ± 0.3 | 5.0 ± 0.5 | 6.5 ± 0.4 |

| **Female Parent Behaviors** | | **Self-care** | |
| --- | --- | --- | --- |
| # of observations | | *Self-grooming* | *Eating and drinking* |
| *Early Light Phase* | **WT-Control** | 1.5 ± 0.3 | 2.4 ± 0.2 |
|  | **HET-*Kmo^+/-^*** | 1.5 ± 0.5 | 3.3 ± 0.4 |
| *Late Light Phase* | **WT-Control** | 1.3 ± 0.2 | 4.2 ± 0.7 |
|  | **HET-*Kmo^+/-^*** | 1.3 ± 0.2 | 3.0 ± 0.3 |

| **Female Parent Behaviors** | | **Negative Behaviors** | |
| --- | --- | --- | --- |
| # of observations | | *Nest maintenance* | *Parent out of nest* |
| *Early Light Phase* | **WT-Control** | 1.7 ± 0.3 | 3.3 ± 1.0 |
|  | **HET-*Kmo^+/-^*** | 1.8 ± 0.8 | 4.4 ± 1.1 |
| *Late Light Phase* | **WT-Control** | 1.0 ± 0 | 4.3 ± 0.8 |
|  | **HET-*Kmo^+/-^*** | 1.7 ± 0.2 | 4.1 ± 0.5 |

| **Male Parent Behaviors** | | **Positive Behaviors** | |
| --- | --- | --- | --- |
| # of observations | | *Grooming pups* | *Contact with pups* |
| *Early Light Phase* | **WT-Control** | 1.0 ± 0 | 7.1 ± 0.5 |
|  | **HET-*Kmo^+/-^*** | 1.5 ± 0.5 | 8.0 ± 0.7 |
| *Late Light Phase* | **WT-Control** | 1.8 ± 0.3 | 8.9 ± 0.4 |
|  | **HET-*Kmo^+/-^*** | 1.1 ± 0.1 | 8.6 ± 0.2 |

| **Male Parent Behaviors** | | **Self-care** | |
| --- | --- | --- | --- |
| # of observations | | *Self-grooming* | *Eating and drinking* |
| *Early Light Phase* | **WT-Control** | 1.5 ± 0.2 | 3.0 ± 0.3 |
|  | **HET-*Kmo^+/-^*** | 1.5 ± 0.3 | 2.4 ± 0.5 |
| *Late Light Phase* | **WT-Control** | 1.5 ± 0.2 | 2.3 ± 0.5 |
|  | **HET-*Kmo^+/-^*** | 1.8 ± 0.2 | 2.0 ± 0.3 |

| **Male Parent Behaviors** | | **Negative Behaviors** | |
| --- | --- | --- | --- |
| # of observations | | *Nest maintenance* | *Parent out of nest* |
| *Early Light Phase* | **WT-Control** | 1.8 ± 0.4 | 4.3 ± 0.6 |
|  | **HET-*Kmo^+/-^*** | 1.7 ± 0.7 | 3.7 ± 0.6 |
| *Late Light Phase* | **WT-Control** | 1.6 ± 0.5 | 4.1 ± 1.0 |
|  | **HET-*Kmo^+/-^*** | 1.4 ± 0.3 | 2.5 ± 0.2 |

**Supplementary Figure 4. Impaired learning in female wild-type (WT-*Kmo^+/+^*) offspring from HET-*Kmo^+/-^*parents.**

**(A)** Latency in the Barnes maze (Females: Two-way RM ANOVA Genotype effect F_(1, 36)_= 5.004, ^P<0.05 with Bonferroni’s post hoc test *P<0.05).

**(B)** Percent of time immobile in the reversal Barnes maze.

Data are mean ± SEM. N = 19-21 per group.
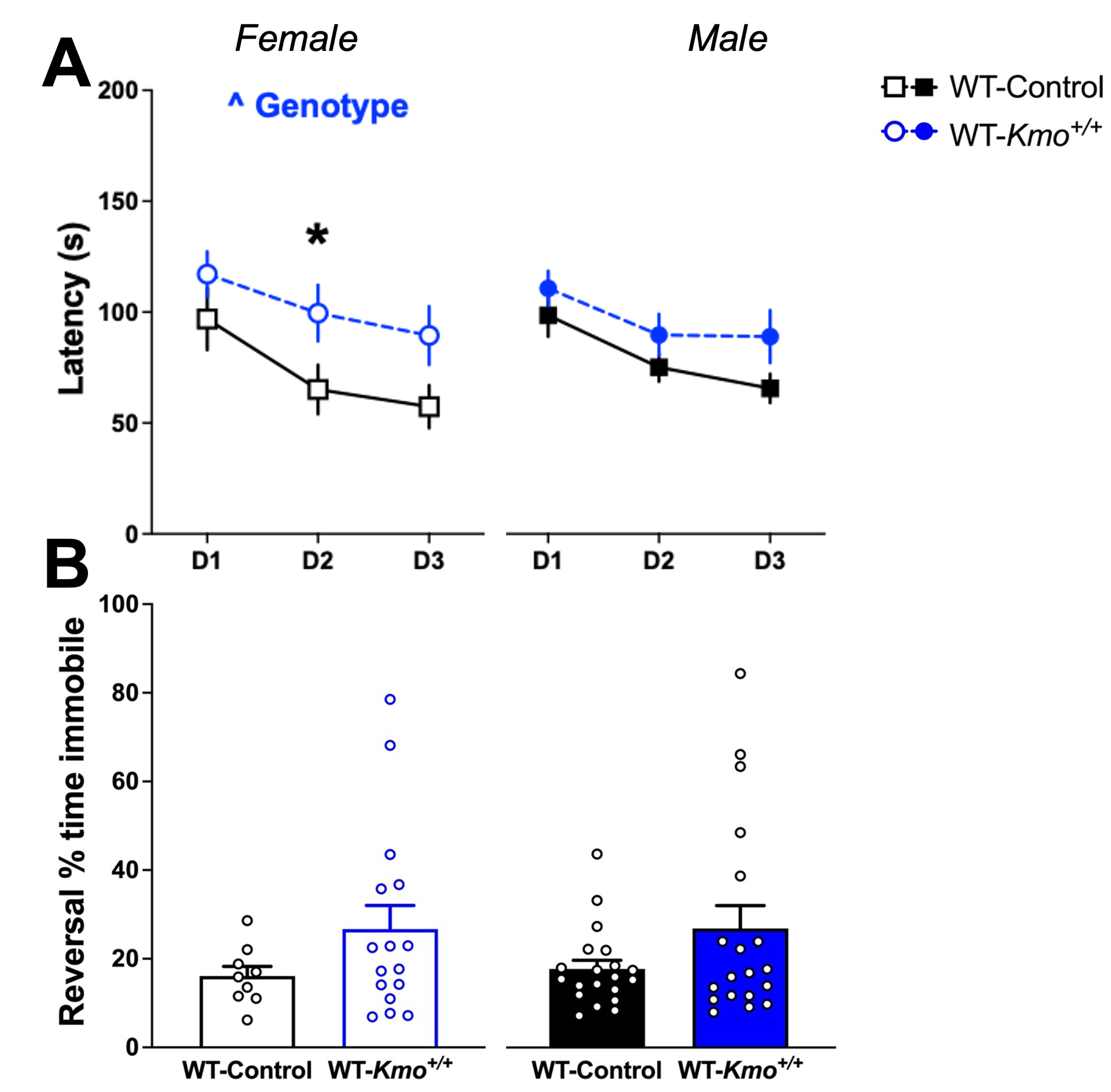


**Supplementary Table 4. Barnes maze search strategies in WT-*Kmo^+/+^* mice in comparison to wild-type (WT-Control) mice.** Data represent % of total number of utilized search strategies. N=19-21 per group.

|  | **Search Strategy** | **Female** | | **Male** | |
| --- | --- | --- | --- | --- | --- |
|  |  | WT-Control | WT-*Kmo^+/+^* | WT-Control | WT-*Kmo^+/+^* |
|  | Direct (% total) | 5 | 0 | 7.76 | 2.50 |
| Day 1 | Serial (% total) | 33.64 | 30.56 | 15.66 | 38.29 |
|  | Random (% total) | 61.36 | 69.44 | 76.58 | 59.21 |
|  | Direct (% total) | 5.57 | 8.33 | 0 | 7.54 |
| Day 2 | Serial (% total) | 71.83 | 43.42 | 45 | 57.14 |
|  | Random (% total) | 22.60 | 48.25 | 55 | 35.32 |
|  | Direct (% total) | 10.53 | 7.89 | 7.89 | 5.28 |
| Day 3 | Serial (% total) | 81.58 | 55.26 | 52.63 | 73.61 |
|  | Random (% total) | 7.89 | 36.84 | 39.47 | 21.11 |
|  | Direct (% total) | 16.99 | 13.89 | 28.03 | 15.13 |
| Reversal | Serial (% total) | 71.57 | 54.17 | 53.95 | 66.71 |
|  | Random (% total) | 11.44 | 31.94 | 18.03 | 18.16 |

**Supplementary Table 5. Sleep-wake parameters in WT-*Kmo^+/+^* mice in comparison to wild-type (WT-Control) mice during light and dark phase.** Data are mean ± SEM. Unpaired t test: *P<0.05 vs. WT-Control. N = 6-10 per group.

| **Light Phase** | **Female** | | **Male** | |
| --- | --- | --- | --- | --- |
|  | WT-Control | WT-*Kmo^+/+^* | WT-Control | WT-*Kmo^+/+^* |
| Total Sleep Duration (min) | 448 ± 9 | ***408 ± 14 **** | 446 ± 10 | 434 ± 8 |
| REM Duration (min) | 40 ± 3 | 35 ± 3 | 41 ± 2 | 43 ± 2 |
| REM Bouts (#) | 35 ± 3 | 31 ± 2 | 36 ± 2 | 34 ± 2 |
| NREM Bouts (#) | 111 ± 7 | 114 ± 6 | 121 ± 8 | 107 ± 3 |
| Wake Bouts (#) | 107 ± 6 | 113 ± 6 | 118 ± 9 | 104 ± 3 |
| REM Bout Duration (s/bout) | 71 ± 2 | 69 ± 2 | 75 ± 2 | 81 ± 2 |
| NREM Bout Duration (s/bout) | 235 ± 19 | 208 ± 7 | 227 ± 29 | 237 ± 10 |
| Wake Bout Duration (s/bout) | 196 ± 14 | 235 ± 35 | 189 ± 16 | ***238 ± 15 **** |

| **Dark Phase** | **Female** | | **Male** | |
| --- | --- | --- | --- | --- |
|  | WT-Control | WT-*Kmo^+/+^* | WT-Control | WT-*Kmo^+/+^* |
| Total Sleep Duration (min) | 265 ± 20 | 262 ± 16 | 281 ± 16 | 277 ± 12 |
| REM Duration (min) | 17 ± 1 | 19 ± 2 | 21 ± 1 | 20 ± 1 |
| NREM Duration (min) | 248 ± 20 | 242 ± 16 | 260 ± 15 | 257 ± 11 |
| Wake Duration (min) | 455 ± 20 | 458 ± 16 | 439 ± 16 | 443 ± 12 |
| REM Bouts (#) | 15 ± 1 | 17 ± 1 | 18 ± 1 | 18 ± 1 |
| NREM Bouts (#) | 71 ± 5 | 72 ± 6 | 92 ± 6 | 80 ± 5 |
| Wake Bouts (#) | 74 ± 5 | 76 ± 5 | 93 ± 6 | 82 ± 5 |
| REM Bout Duration (s/bout) | 77 ± 5 | 70 ± 2 | 72 ± 3 | 67 ± 3 |
| NREM Bout Duration (s/bout) | 223 ± 24 | 202 ± 3 | 178 ± 12 | 196 ± 11 |
| Wake Bout Duration (s/bout) | 769 ± 92 | 770 ± 128 | 619 ± 127 | 688 ± 61 |

**Supplementary Figure 5. Sex-specific differences in NREM and REM sleep power spectra in WT-*Kmo^+/+^* compared to wild-type (WT-Control) mice during the dark phase.**

**(A)** NREM sleep spectral power (Females: Two-way RM ANOVA Genotype x Frequency interaction F_(38, 380)_= 1.821, ^^P<0.01).

**(B)** REM sleep spectral power (Males: Two-way RM ANOVA Genotype effect F_(1, 16)_= 4.676, ^P<0.05).

Data are mean ± SEM. N = 6-10 per group.
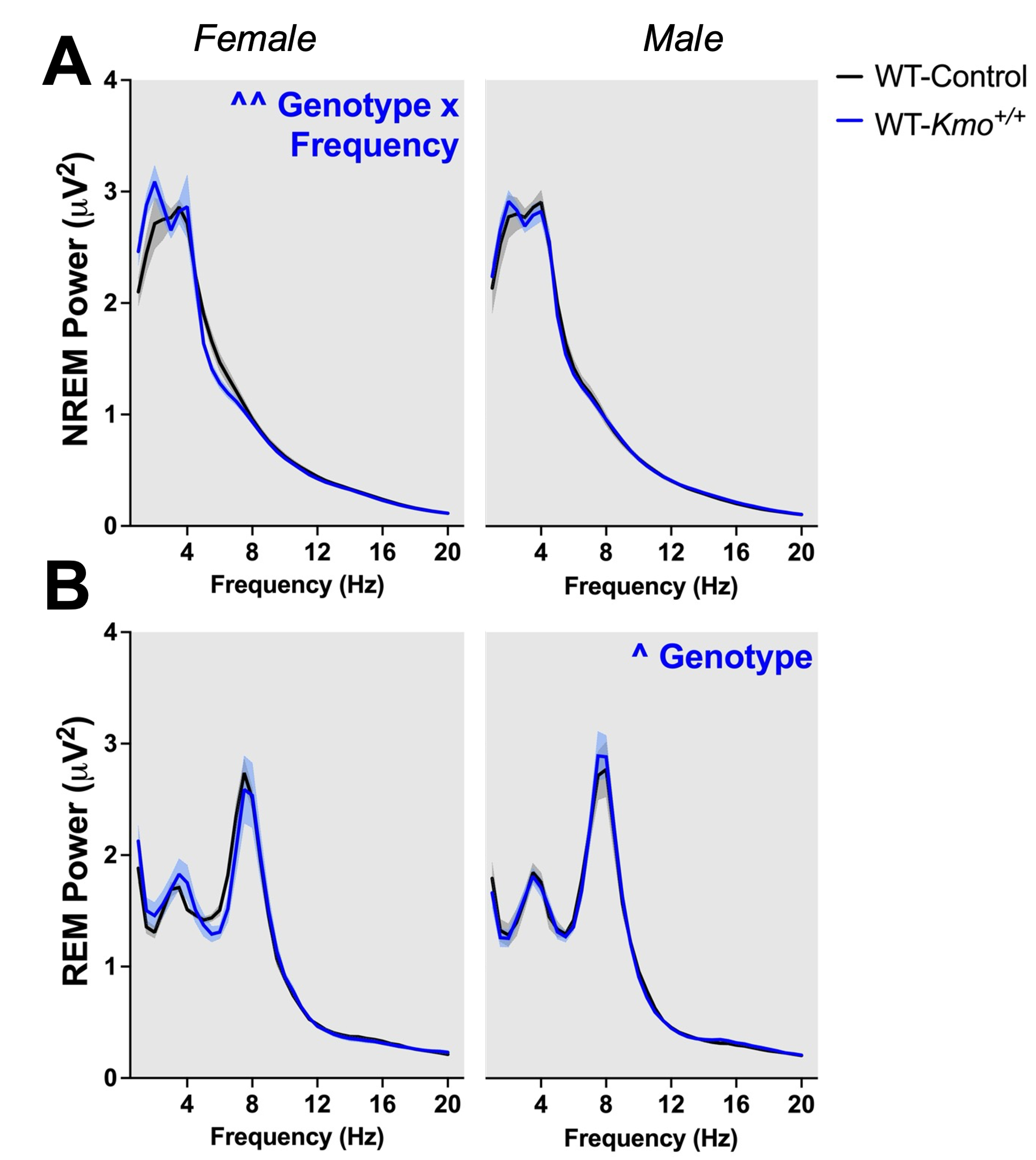

Supplement: Supplementary file 1 — Supplementary Material 1. [file 13293_2025_703_MOESM1_ESM.docx]
